# The Oligonucleotide Binding (OB)-Fold Domain of KREPA4 Is Essential for Stable Incorporation into Editosomes

**DOI:** 10.1371/journal.pone.0046864

**Published:** 2012-10-04

**Authors:** Smriti Kala, Houtan Moshiri, Vaibhav Mehta, Chun Wai Yip, Reza Salavati

**Affiliations:** 1 Institute of Parasitology, McGill University, Ste. Anne de Bellevue, Quebec, Canada; 2 Department of Biochemistry, McGill University, Montreal, Quebec, Canada; 3 McGill Centre for Bioinformatics, McGill University, Montreal, Quebec, Canada; NIGMS, NIH, United States of America

## Abstract

Most mitochondrial mRNAs in trypanosomatid parasites require uridine insertion/deletion RNA editing, a process mediated by guide RNA (gRNA) and catalyzed by multi-protein complexes called editosomes. The six oligonucleotide/oligosaccharide binding (OB)-fold proteins (KREPA1-A6), are a part of the common core of editosomes. They form a network of interactions among themselves as well as with the insertion and deletion sub-complexes and are essential for the stability of the editosomes. KREPA4 and KREPA6 proteins bind gRNA *in vitro* and are known to interact directly in yeast two-hybrid analysis. In this study, using several approaches we show a minimal interaction surface of the KREPA4 protein that is required for this interaction. By screening a series of N- and C-terminally truncated KREPA4 fragments, we show that a predicted α-helix of KREPA4 OB-fold is required for its interaction with KREPA6. An antibody against the KREPA4 α-helix or mutations of this region can eliminate association with KREPA6; while a peptide fragment corresponding to the α-helix can independently interact with KREPA6, thereby supporting the identification of KREPA4-KREPA6 interface. We also show that the predicted OB-fold of KREPA4; independent of its interaction with gRNA, is responsible for the stable integration of KREPA4 in the editosomes, and editing complexes co-purified with the tagged OB-fold can catalyze RNA editing. Therefore, we conclude that while KREPA4 interacts with KREPA6 through the α-helix region of its OB-fold, the entire OB-fold is required for its integration in the functional editosome, through additional protein-protein interactions.

## Introduction

Mitochondrial RNA editing in trypanosomes is a form of post-transcriptional RNA processing that creates mature functional mRNAs by insertion and deletion of uridylates (Us) into mitochondrial mRNAs, as specified by gRNAs (reviewed in [1]–[3]). Each gRNA specifies the editing of several sites and multiple gRNAs are used to edit most mRNAs [4]. While each gRNA has a distinct sequence, they all have a conserved secondary structure, consisting of two stem/loop regions at the 5′ end, named stem/loop I and II, and a 3′ oligo(U)-tail [5], [6]. These structural elements of the guide either specify the interaction between the gRNA and its cognate mRNA or play a role in stabilization of the gRNA/pre-mRNA duplex [4], [7], [8].

RNA editing is catalyzed by multi-protein complexes, the 20S editosomes; that sediment at ∼20S on glycerol gradients and contain the four key enzyme activities that cleave the mRNA, insert or delete Us and ligate the edited products (reviewed in [9]–[11]). The number of proteins in the fully functional editosome is not known; however, the most recent studies have identified around 20 proteins (Kinetoplastid RNA Editing Proteins or KREPs) that have predicted catalytic and/or RNA interaction motifs [10], [12]. While several different nomenclatures have been proposed to designate the editosome proteins, their differences have been described in [13]. Here we have followed the nomenclature proposed by Stuart et al. [10].

Other complexes involved in RNA editing include the MRP1/MRP2 complex, which has a matchmaking type of RNA annealing activity [14]–[16]. RBP16 also plays a role in gRNA/pre-mRNA interaction, and has an overlapping function with MRP1 and MRP2 proteins [17], [18]. The Mitochondrial RNA Binding complex 1 (MRB1) or the Guide RNA Binding Complex (GRBC), has recently been described to play a central role in coordinating diverse aspects of mitochondrial RNA metabolism such as RNA editing, stability, polyadenylation and translation [19]–[28].

Three distinct forms of the 20S editosomes exist, all with a common set of 12 core proteins but each one is associated with a different endonuclease [29]–[32]. The six related OB-fold proteins (KREPA1-A6), out of which the three largest (KREPA1-A3) also contain two N-terminally conserved zinc-finger (Zf) motifs, are part of the common core of 20S editosomes [12], [33], [34]. OB-fold domains and Zf motifs function in nucleic acid recognition and/or protein binding [35]–[37]. Recent data propose that extensive protein–protein interactions mediated by OB-fold proteins are essential for the structural integrity and functioning of the 20S editosomes [38].

Within the core complex, KREPA1 and KREPA2 form two distinct catalytic sub-complexes involved in insertion and deletion editing activities, respectively. KREPA1 associates with KRET2 3′ terminal uridylyl transferase (TUTase) and KREL2 RNA ligase, resulting in the insertion sub-complex with U insertion and ligation activities [34]. Similarly, KREPA2 associates with KREX2 3′ exonuclease and KREL1 RNA ligase, resulting in the deletion sub-complex with U removal and ligation activities. Both KREPA1 and KREPA2 proteins are critical for the assembly of the 20S core complex, in which KREPA1 interacts with KREPA6 and KREPA2 interacts with both KREPA3 and KREPA6 [34], [38]. Down-regulation of KREPA1 results in preferential inhibition of insertion editing and loss of KREL2 [39], [40], while inactivation of KREPA2 results in loss of KREL1 [41]. KREPA3 and KREPA6 are key components in the interaction network of the 20S editosome, as both can interact with multiple other partners in the complex. KREPA3 can directly interact with KREPA2, KREPA6 and KREPB5 RNase III-like protein, in addition to substrate RNA [38], [42]. While RNAi knockdown of KREPA3 in *T. brucei* Procyclic Form (PF) leads to partial disruption of the 20S editosomes and loss of endonuclease activity, the more extensive repression of KREPA3 expression in *T. brucei* Bloodstream Form (BF) by regulatable knockout, results in complete loss of 20S editosomes as well as *in vitro* and *in vivo* RNA editing activities [43]. ZF motifs of KREPA3 are essential for growth and RNA editing *in vivo*, while the OB-fold domain is necessary for protein interaction as its absence results in loss of editosomes. KREPA6 is a multifunctional protein, critical for the structural integrity of 20S editosomes. It interacts directly with KREPA1 and KREPA2, thereby bridging the insertion and deletion sub-complexes [34], [38]. It also interacts directly with KREPA3 and KREPA4 proteins, and binds gRNAs and pre-edited mRNAs with medium affinity [38], [44]. Knockdown of its expression by RNAi leads to disruption and ultimate loss of 20S editosomes, and strongly affects RNA editing *in vivo* and normal growth of *T. brucei* PF.

KREPA4, another member of KREPA protein family is essential for RNA editing, 20S editosome structural integrity, and normal growth of *T. brucei* PF [45]. It has a predicted OB-fold at the C-terminus and two predicted low compositional complexity regions (LCRs) at the N-terminus. KREPA4 is a gRNA-binding protein with preferential binding specificity toward the 3′ oligo(U) tail. The potential RNA binding surface of KREPA4 is mainly localized to the OB-fold domain, which mediates a high-affinity binding interaction with the gRNA oligo(U) tail, which is the major binding determinant [46]. The N-terminal LCRs further stabilize the binding by sequence-specific interactions with the gRNA stem–loop elements. The OB-fold of KREPA4 also has RNA annealing activity, as it stimulates duplex formation between editing associated gRNA/pre-mRNA pairs *in vitro*. The only editosome protein, with which KREPA4 is known to have a direct interaction, is KREPA6. A yeast two hybrid screen using full-length KREPA6 as bait identified an interacting fragment of KREPA4, which comprised of the full length protein minus the first 25 N-terminal residues (KREPA4^26–218^) [38]. Here in this study, by creating various N- and C-terminally truncated deletion mutants of KREPA4, we have mapped the interaction interface to around 17 amino acid residues (KREPA4^153–169^), comprising an α-helix in the predicted OB-fold domain of KREPA4. In support of our finding, we further demonstrate that this interaction is blocked by an antibody against the predicted KREPA4 α-helix, and mutation of residues in the helix region can similarly inhibit the interaction with KREPA6. We also show that a peptide fragment corresponding to this α-helix can independently interact with KREPA6. Furthermore, we have identified the OB-fold; independent of its gRNA binding activity, as the essential domain for stable integration of KREPA4 in 20S editosomes, and shown that editing complexes co-purified with the tagged OB-fold domain can catalyze *in vitro* RNA editing. Hence, while the α-helix region of KREPA4 OB-fold mediates the direct interaction with KREPA6, the entire domain is required for efficient incorporation of KREPA4 into the editing complexes, possibly through other uncharacterized protein interactions.

## Results

### KREPA4 Binds Directly to KREPA6

**Figure 1:**
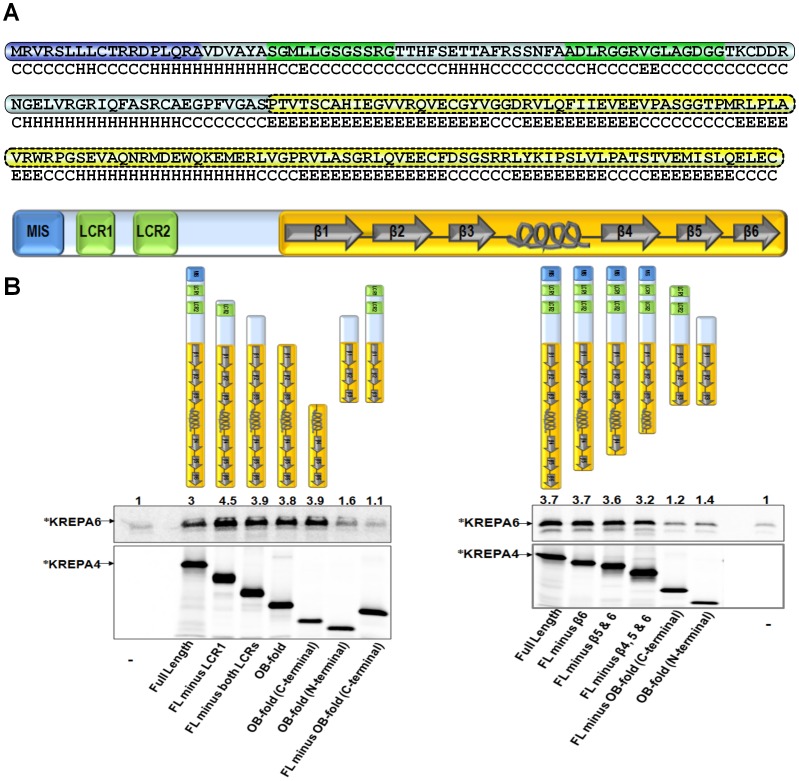
α-helix of KREPA4 OB-fold mediates the interaction with KREPA6. *(A)* Complete amino acid sequence of KREPA4 is shown, with the putative mitochondrial import signal (MIS) sequence highlighted in blue, the predicted low compositional complexity regions (LCRs) in green, and the predicted OB-fold in yellow. The predicted secondary structure of KREPA4 is denoted below the primary amino acid sequence. H stands for α helix, E for β strand, and C for coiled regions. The domain structure of the full length protein is represented schematically; the predicted secondary structure of the OB-fold has been indicated, where arrows represent β strands and the spiral represents the α-helix. *(B, Upper left & right panels*) CoIP of ^35^S-methionine labelled (*) KREPA6 in the presence of the indicated, unlabelled full length or truncated KREPA4 proteins, also represented schematically. An antibody against the Xpress tag on KREPA4 constructs was used for immunoprecipitation. Numbers indicate the level of co-immunoprecipitated KREPA6 relative to the background, which is normalized to 1. Lanes marked with (−) in the bottom panels show the level of KREPA6 retained by non-specific binding to the beads, in the absence of KREPA4. *(B, Lower left & right panels*) Equimolar amounts of ^35^S-methionine labelled (*) KREPA4 constructs present in the CoIP are shown. When both proteins were present together, only one was radiolabelled at a time, as indicated.

A previous report demonstrated that KREPA4 and KREPA6 interact with each other, on the basis of a yeast two-hybrid screen using full-length KREPA6 as bait, which identified KREPA4^26–218^ fragment [38] comprising the full-length protein without the first twenty-six amino acids (Figure 1A). We first confirmed this direct interaction by Coimmunoprecipitation (CoIP) experiments, in which both proteins were ^35^S-methionine labelled and expressed by the *in-vitro* TNT system. While both proteins carried a His-tag; which facilitated a His-tag based quantitation, only KREPA4 carried an exclusive Xpress-tag, on the basis of which the interaction was determined. Due to the similar size of the two proteins, a distinct band could not be visualized for the co-immunoprecipitated product (data not shown), and hence the CoIP had to be carried out with only one protein radiolabelled at one time. The upper panels of Figure 1B show that labelled KREPA6 is pulled down in the presence of full length KREPA4 and this specific interaction is much stronger than the background immunoprecipitation of KREPA6 in the absence of KREPA4 (compare lanes labelled Full Length and -). These data confirm that KREPA4 and KREPA6 directly interact with each other.

### A Predicted Helical Region of KREPA4 OB-fold Mediates the Interaction with KREPA6

To map the KREPA4 region responsible for interaction with KREPA6, we created various KREPA4 truncations that progressively removed the predicted structural motifs from either the N-terminal or C-terminal regions of KREPA4, and are named after the predicted motifs that are retained. These structural motifs and the basis of their prediction have been described in a previous report [46]. Since a predicted S1-motif was found not to occur in KREPA4, a revised schematic representation of the domain structure of full length KREPA4, with secondary structure prediction of the OB-fold is illustrated in Figure 1A.

All KREPA4 truncations carried the Xpress-tag and expressed well with the TNT system (Figure 1B, lower left panel). It should be noted that *equimolar* concentrations of various KREPA4 truncations were used; therefore the larger proteins appear more intense on the gel owing to their higher molecular masses (Figure 1B, lower left panel). Secondly, as the proteins were labelled with ^35^S-methionine, the larger truncations with additional methionines generally appear more intense. Figure 1B, upper left panel shows that all the KREPA4 truncations that retained the C-terminal region of the predicted OB-fold (α-helix and β-strands 4, 5, and 6), were able to pull down KREPA6 even more efficiently than the full length KREPA4. On the other hand, the OB-fold (N-terminal) and FL minus OB-fold (C-terminal) truncations, lacking the C-terminal region of the OB-fold domain pulled down KREPA6 only marginally above the background level. These data suggest that the C-terminal region of KREPA4 OB-fold, comprising the α-helix and β-strands 4, 5, and 6, mediates the interaction with KREPA6.

**Figure 2:**
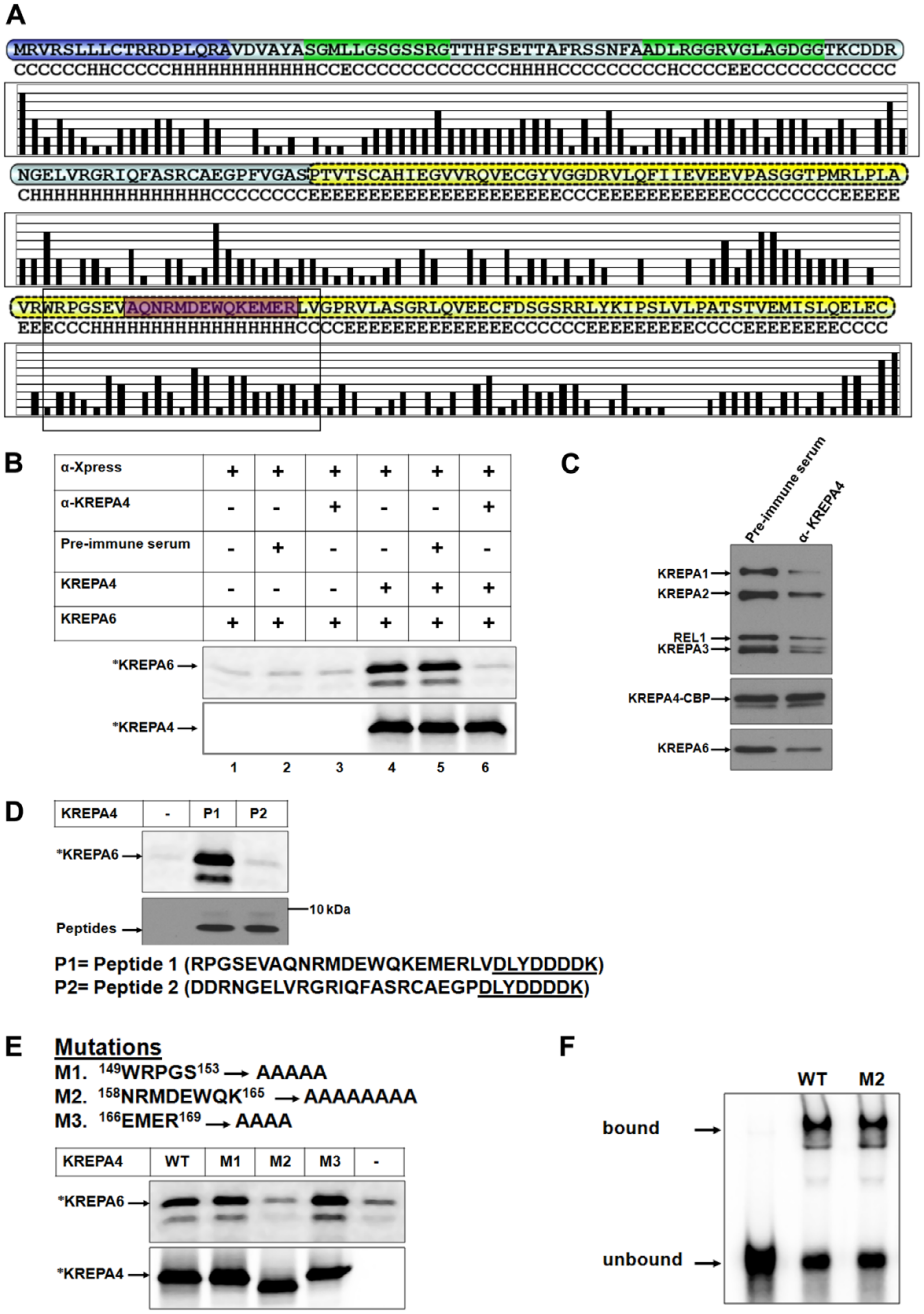
An antibody against the α-helix, or mutagenesis of this region inhibits the interaction with KREPA6. *(A)* Solvent accessibility prediction of residues in KREPA4 secondary structure. Higher bars indicate hydrophilic exposed residues while lower bars indicate hydrophobic buried residues. The α-helix of KREPA4 OB-fold, hypothesized to mediate interaction with KREPA6 is highlighted by a box. The peptide sequence used for generating anti-KREPA4 antibody is highlighted in purple. *(B, upper panel)* CoIP of ^35^S-methionine labelled (*) KREPA6 with unlabelled full length KREPA4, using antibody against the KREPA4 Xpress tag (α -Xpress). Table indicates the presence or absence of the α-KREPA4 antibody and the pre-immune serum, as well as whether proteins are present singly or mixed together. *(B, lower panel)* The amount of ^35^S-methionine labelled (*) KREPA4 present in the CoIP is shown. When both proteins were present together, only one was radiolabelled at a time, as indicated. *(C)* The TEV eluate from KREPA4 TAP-tagged cells was treated with anti-KREPA4 antibody (α-KREPA4) or pre-immune serum as indicated, subjected to calmodulin affinity purification and eluates analysed by western blotting with antibodies as indicated. (*D, upper panel*) CoIP of ^35^S-methionine labelled (*) KREPA6 in the presence of KREPA4 peptide fragments as indicated. Lane marked with (−) shows the level of KREPA6 retained by non-specific binding to the beads, in the absence of KREPA4 peptides. (*D, lower panel*) The amounts of KREPA4 peptides present in the CoIP, as determined by western analysis with anti-Xpress antibody. The sequences of both peptides are indicated. Presence of the Xpress tag (underlined) on both peptides enabled the use of anti-Xpress antibody for the CoIP. *(E)* Summary of the KREPA4 α-helix mutations: the mutated residue positions are indicated. *Upper panel* CoIP of ^35^S-methionine labelled (*) KREPA6 in the presence of unlabeled wild-type (WT) or mutant (M) full length KREPA4 using antibody against the KREPA4 Xpress tag. Lane marked with (−) shows the level of KREPA6 retained by non-specific binding to the beads, in the absence of KREPA4. *Lower panel* Equimolar amounts of ^35^S-methionine labelled (*), wild-type or mutated KREPA4 proteins present in the CoIP are shown. When both proteins were present together, only one was radiolabelled at a time, as indicated. In case of M2, the alanine substitution has changed the overall charge and therefore, the electrophoretic migration of this mutant in comparison to the wild-type KREPA4. *(F)* Gel shift assay to monitor the gRNA-binding activity of equal amounts of wild-type (WT) and mutant (M2) KREPA4 proteins. The bound and free radiolabelled gA6 [14] gRNA is indicated.

To further define the C-terminal region of KREPA4 OB-fold which mediates the interaction with KREPA6, a set of C-terminal deletion mutants of KREPA4, progressively removing predicted β-strands 6, 5, and 4 was generated. These truncations also carried the Xpress-tag and expressed well with the TNT system (Figure 1B, lower right panel). CoIP experiments using equimolar amounts of KREPA6 and full length KREPA4 or the three C-terminal truncations, co-immunoprecipitated similar levels of KREPA6 (Figure1B, upper right panel). While the smallest C-terminal truncation lacking β-strands 4, 5, and 6, still retained the interaction with KREPA6, the truncation lacking the entire C-terminal region of the OB-fold (α-helix and β-strands 4, 5, and 6) lost this interaction. The only difference between these two truncations is that the construct which maintained the interaction with KREPA6, retained the α-helix, connecting β strands 3 and 4 of the predicted OB-fold. According to solvent accessibility predictions, a number of polar and charged as well as a few hydrophobic residues are present in this helix region (Figure 2A), possibly forming the interface of interaction with KREPA6. This possibility was further investigated by the experiments described in the following sections.

### Antibody Against the α-helix of KREPA4 OB-fold Blocks the Interaction with KREPA6

We generated a polyclonal antibody raised against a synthetic peptide that overlaps with the α-helix of the OB-fold domain of KREPA4 (Figure 2A), which according to our hypothesis is the region that mediates the interaction with KREPA6. Hence, this antibody should be able to block the KREPA4-KREPA6 interaction, if indeed the interaction is mediated by the proposed α-helix of KREPA4. To test our hypothesis, CoIP experiments were performed in which KREPA4 and KREPA6 were incubated in the presence of the anti-KREPA4 antibody or the pre-immune serum as a control. As shown in Figure 2B, upper panel, KREPA6 failed to co-immunoprecipitate in presence of the anti-KREPA4 antibody (compare lanes 4 and 6), whereas the pre-immune serum did not have any inhibitory effect on the pull down of KREPA6 (compare lanes 4 and 5). Figure 2B, lower panel shows that the level of immunoprecipitated KREPA4 is the same between lanes 4, 5 and 6. Hence the anti-KREPA4 antibody was able to obstruct the interaction of the two proteins, which verifies that the α-helix in the OB-fold of KREPA4 mediates the interaction with KREPA6.

Next, we examined the effect of the anti-KREPA4 antibody, which can block the KREPA4-KREPA6 interaction, on the integrity of the editosome complex. Using *T. brucei* cell line which expresses tandem affinity purification (TAP)-tag of full length KREPA4, editosome was obtained through the first TEV purification step and the eluate was split in half. One half of the TEV eluate was incubated with the anti-KREPA4 antibody, while the other half was incubated with the pre-immune serum as a control. Treated samples were then bound to the calmodulin column, and the eluted fractions were pooled and subjected to western blot analysis to compare the amounts of editosome proteins in the two samples. As shown in Figure 2C, while the levels of tagged KREPA4 remained unchanged, a significant reduction was observed in the amounts of all other editosome proteins tested, which implies that the anti-KREPA4 antibody could obstruct the KREPA4-KREPA6 interaction, resulting in partial dissociation of some KREPA4 proteins from the editosome complex. As KREPA4 is essential for structural integrity of editosomes, these data suggest that partial dissociation of KREPA4 results in an overall reduction in the amounts of the editosome proteins. Considering that there are three editosomes with three different endonucleases; which may exchange subunits, the interactions among the editosome complexes seem to be dynamic *in vitro*, and therefore the antibody with a high affinity for KREPA4 was able to partially disrupt these interactions. Similar experiments demonstrating the inhibition of spliceosome formation, by using antibodies against different splicing factors have been reported [47], [48]. Hence inhibition of the KREPA4-KREPA6 interaction affects complex stability and/or integrity. However, this inhibition did not result in a complete loss of the editosome proteins because KREPA4 may still remain associated with the complex owing to its interaction with gRNA and/or other proteins. Whether or not, the interaction with gRNA is essential for incorporation of KREPA4 into editing complexes was tested, and has been described in a later section.

### A Peptide Fragment Corresponding to the α-helix of KREPA4 OB-fold can Mediate Interaction with KREPA6

In order to test more conclusively, the role of this predicted helical region of KREPA4 OB-fold, in interaction with KREPA6, we synthesized two peptide fragments. While Peptide#1 corresponds to the α-helix *within* the OB-fold of KREPA4, a control Peptide #2 corresponds to another predicted helical region *outside* the OB-fold. Both peptides carried the Xpress tag, which enabled their pull-down by beads conjugated to anti-Xpress antibody. When equal concentrations of these peptides were used, radiolabelled KREPA6 only coimmunoprecipitated with Peptide#1 (Figure 2D). This clearly demonstrates that residues comprising the α-helix region of KREPA4 OB-fold, mediate the interaction with KREPA6.

### Mutagenesis of Residues in the α-helix of KREPA4 OB-fold Inhibits the Interaction with KREPA6

As an alternative approach to validate the role of the α-helix of KREPA4 OB-fold in interaction with KREPA6, we made mutations of a few residues in and around the predicted α-helix region. These mutations are summarized in Figure 2E, where mutant 1 represents a control; in which residues in the predicted loop region just before the helix have been mutated, whereas in mutants 2 & 3, residues within the predicted α-helix region have been mutated. CoIP experiments with equimolar amounts of wild-type and mutant full length KREPA4 proteins showed that mutant 2 had lost the interaction with KREPA6, whereas mutants 1 and 3 interacted with KREPA6 as efficiently as wild-type KREPA4 (Figure 2E, upper panel). In mutant 2, a stretch of eight amino acids was substituted by alanines, which changed the overall charge and therefore, the electrophoretic migration of this mutant in comparison to the wild-type KREPA4 (Figure 2E, lower panel). Six out of the eight mutated residues were polar or charged, while two were hydrophobic, and therefore these residues could establish electrostatic and hydrophobic interactions with KREPA6. A multiple sequence alignment of the predicted OB-folds of all KREPA proteins shows that some of these residues are highly conserved, such as N^158^ is at a conserved polar position, M^160^ is at a conserved hydrophobic position, E^162^ is conserved between KREPA4 and KREPA6 and W^163^ is at a conserved hydrophobic position [12].

To demonstrate that the loss of KREPA6 binding capacity was not caused by misfolding of the mutated protein, we compared the RNA-binding potential of the wild-type and mutant KREPA4 proteins. As shown in Figure 2F, mutant 2, displayed as efficient RNA-binding activity as the wild-type KREPA4, and therefore the loss of interaction with KREPA6 was not due to a grossly misfolded protein. Therefore, based on these data, once again we conclude that the α-helix of the KREPA4 OB-fold mediates the interaction with KREPA6.

### The OB-fold of KREPA4 is Required for its Integration into the Editosomes

**Figure 3:**
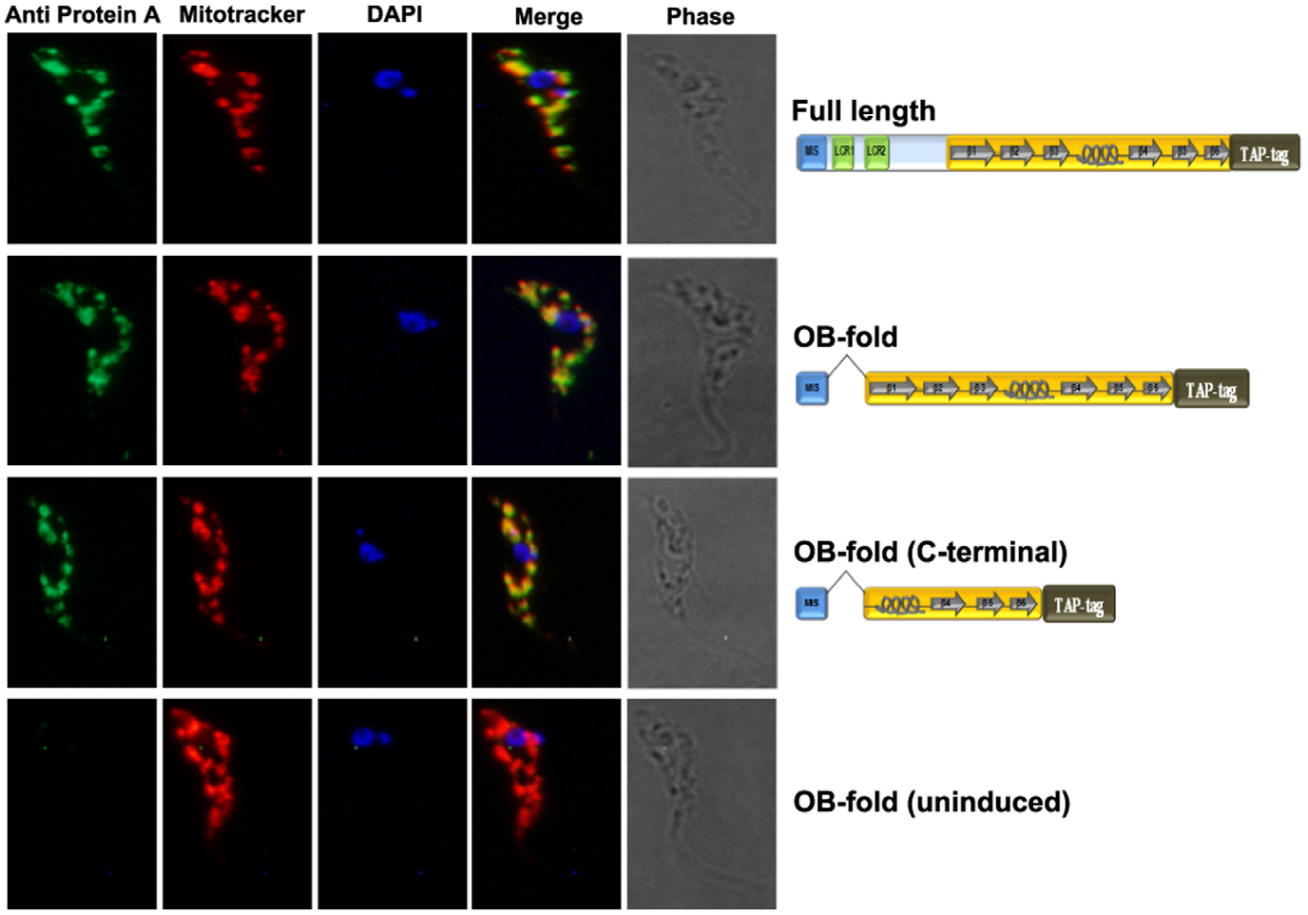
Mitochondrial localization of TAP-tagged full length and truncated KREPA4 proteins. TAP-tagged full length and truncated KREPA4 proteins were detected in the mitochondria as revealed by co-localization with anti protein-A antibody and mitotracker stain. DAPI was used for staining of the nucleus and kinetoplast. An uninduced control, not expressing the TAP-tag did not show any staining with the anti-protein A antibody.

Next, we investigated if the interaction with KREPA6 is the only interaction by means of which KREPA4 gets incorporated into the 20S editosomes. To assess which domain of KREPA4 is responsible for its integration into the editosome, we designed TAP-tagged constructs of the majority of the KREPA4 deletion variants used for the CoIP experiments. The predicted mitochondrial import signal (MIS) was included in all constructs to enable mitochondrial translocation. In the case of two constructs, OB-fold and OB-fold (C-terminal), the MIS was fused with these KREPA4 fragments (Figure 3, schematics). Therefore, in cell lines expressing the tagged proteins, we confirmed the mitochondrial localization of these two constructs by immunofluorescence microscopy, using antibody against the protein-A tag which showed the same pattern of staining as the mitotracker (Figure 3). An uninduced cell line, not expressing the TAP-tag did not show any staining, thereby demonstrating the specificity of the anti-protein A antibody. It must be noted here that the single mitochondrion of *T. brucei* usually shows a characteristic tubular pattern upon mitotracker staining, as opposed to the patchy localization. Such fragmented appearance of the mitochondria has been reported in another study [49], and could possibly be related to cell cycle progression and kDNA replication.

**Figure 4:**
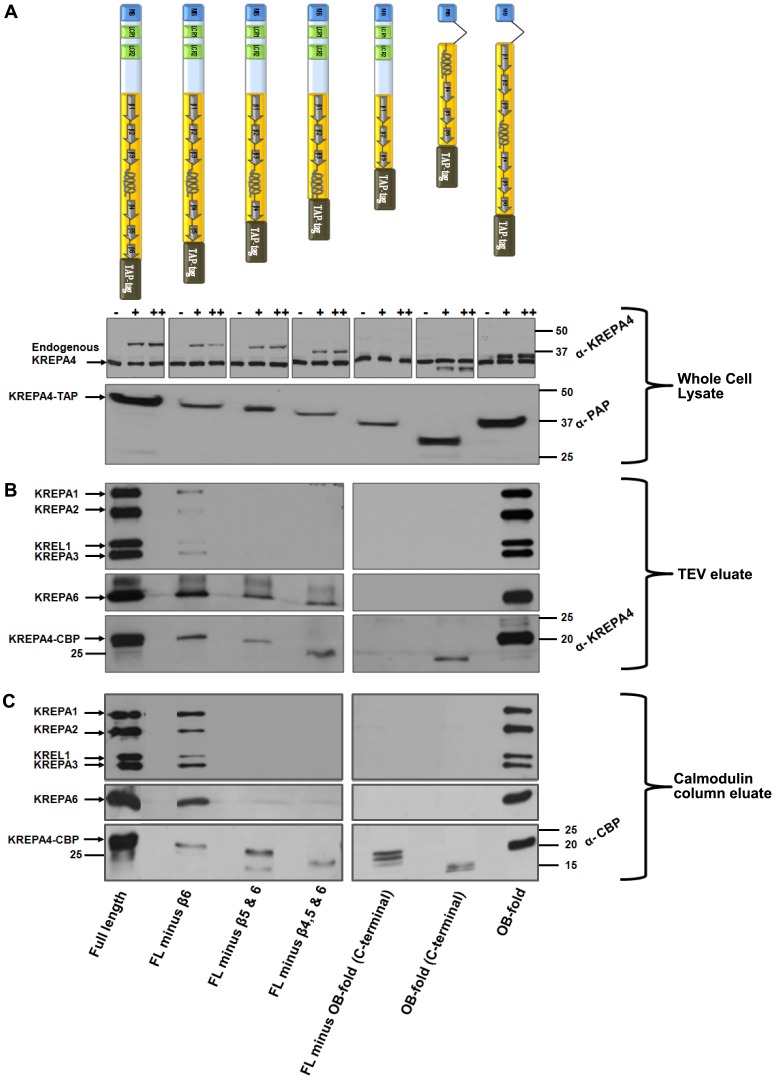
The OB-fold of KREPA4 is required for its integration into the editosomes. *(A, upper panel)* Each TAP-tagged KREPA4 construct is represented schematically. Equivalent numbers of cells from full length or truncated KREPA4 TAP-tag cell lines were lysed, and the total cell lysate was subjected to western analysis. Endogenous and tagged KREPA4 were detected using anti-KREPA4 antibody. (−) indicates absence of tetracycline while (+) and (++) indicate the presence of 100 ng/ml or 1 µg/ml of tetracycline. In case of the FL minus OB-fold (C-terminal) truncation, only the endogenous KREPA4 is visible as the truncation lacks the region recognized by the anti-KREPA4 antibody. The molecular weight markers are indicated. *(A, lower panel)* Equivalent numbers of induced cells were harvested from full length or truncated KREPA4 TAP-tag cell lines and the total lysates were subjected to western analysis. Tagged KREPA4 was detected using PAP reagent against the Protein-A tag. The molecular weight markers are indicated. *(B)* The TEV eluate from full length or truncated KREPA4 TAP-tagged cells was subjected to western analysis with antibodies as indicated. The molecular weight markers are indicated in the bottom panel to show the difference in size between different KREPA4 truncations. As mentioned above, the anti KREPA4 antibody cannot detect the FL minus OB-fold (C-terminal) truncation (bottom panel). *(C)* The TEV eluates were further purified by calmodulin affinity and the pooled eluates (2&3) were subjected to western analysis with antibodies as indicated. The molecular weight markers are indicated in the bottom panel to show the difference in size between different KREPA4 truncations. An antibody against the calmodulin-binding protein (CBP) tag was used in order to detect all the KREPA4 truncations (bottom panel).

After confirming mitochondrial translocation of our TAP tag constructs, we tested these constructs for their ability to associate with editosome complexes. The TAP-tagged KREPA4 full length protein and truncated variants (Figure 4A, schematics), when expressed in the presence of tetracycline did not show any difference in the growth rate of induced versus uninduced cells, or among different truncations (data not shown). Total cell lysate from equivalent number of cells when probed with anti-KREPA4 antibody showed two distinct bands, one corresponding to the endogenous KREPA4 and the other to the TAP-tagged KREPA4 (Figure 4A, upper panel). The level of endogenous KREPA4 did not change upon expression of the tagged KREPA4. However, the expression level of different TAP-tagged KREPA4 truncations varied; the full length and OB-fold constructs were expressed at a higher level than other constructs. The same pattern was observed when the total cell lysate was probed with the PAP reagent, which binds to the protein-A component of the TAP-tag construct (Figure 4A, lower panel). TAP-tagged FL minus OB-fold (C-terminal) truncation could not be visualized with the anti-KREPA4 antibody because it lacks the α-helix region that is recognized by the antibody, however it could be detected with the PAP reagent.

#### (i) Western blot analysis of TEV eluates

As the level of endogenous KREPA4 is similar in all truncations (Figure 4A, upper panel), the tagged constructs would be expected to face the same extent of competition from the endogenous KREPA4 for assembling into the editosomes. In this scenario, editing complexes would co-purify only with the truncations which retained the regions/domains essential for association with the editosomes. Equivalent number of cells expressing the tagged KREPA4, full length or truncated variants, were harvested and subjected to the TAP-tag purification protocol. TEV eluates from the first step of purification were subjected to western blot analysis with monoclonal antibodies (MAbs) against KREPA1, KREPA2, KREPA3 and KREL1, to verify the presence of editosome complexes (Figure 4B, top panel). Besides the full length KREPA4, only two other truncations, FL minus β6 and OB-fold, were able to foster the major interactions with the editosome complexes, although, to different extents. The co-purified editosome proteins were less abundant in the FL minus β6 truncation, compared to the OB-fold and full length KREPA4 constructs. This could be attributed to the lower expression level of the FL minus β6 truncation in the starting material, as evident from the whole cell lysate probed with PAP reagent (Figure 4A, lower panel). Since the two truncations that retained the interactions with the editosomes have the OB-fold in common, this indicates that the predicted OB-fold domain is required for the association of KREPA4 with the editosome complexes. None of the other truncations showed the presence of editosome proteins in their TEV eluates even upon longer exposures of the western blots. This indicates that these truncations were devoid of regions/domain essential for incorporation into the editosome complexes.

Western blot analysis of the TEV eluates with antibody against KREPA6 (Figure 4B, middle panel) showed that the full length KREPA4, FL minus β6 and OB-fold proteins maintained their interaction with KREPA6. This was expected as these constructs also retained the interactions with the editosome complexes, and KREPA6 is a part of the core complex. But unexpectedly, the TEV eluates of truncations FL minus β5 & 6 and FL minus β4, 5 & 6, which did not show the presence of editosome complexes, still showed an interaction with KREPA6. These data suggest direct interaction between these truncations and freely occurring KREPA6 molecules, which may be independent of the 20S editosome complexes. These data are in agreement with our *in vitro* CoIP data which show that all of the above mentioned truncations are capable of a direct interaction with KREPA6 (Figure 1B). In further agreement with the CoIP data, the TEV eluate of FL minus OB-fold (C-terminal) (truncation lacking the α-helix shown to mediate the interaction with KREPA6), does not show the presence of KREPA6. The only discrepancy between the TAP-tag and CoIP data is that KREPA6 could not be detected in the TEV eluate of the OB-fold (C-terminal) construct. This is in contrast to our CoIP data which show a direct interaction of this construct with KREPA6. Failure of mitochondrial translocation cannot possibly be the cause of this inconsistency, because mitochondrial localization of this TAP-tagged construct was confirmed by immunofluorescence microscopy (Figure 3). As this is the smallest KREPA4 fragment fused to the TAP-tag, therefore, the lack of interaction could be attributed to interference from the large tag (20.6 kDa), which might give rise to a contorted configuration of the relatively smaller protein fragment (8.1 kDa). Nonetheless, analysis of data from TAP-tag experiments alone, also points towards the α-helix in the OB-fold of KREPA4 as the region responsible for mediating the interaction with KREPA6. On the other hand, the complete OB-fold domain seems to be essential for incorporation of KREPA4 into the editosomes.

When the TEV eluates from the constructs were probed with anti-KREPA4 antibody (Figure 4B, bottom panel), they showed the same expression profile of the tagged proteins as the total lysates probed with PAP reagent (Figure 4A, lower panel). The full length and OB-fold constructs were expressed at a higher level than all other constructs. Consistent with the expression levels of tagged constructs, the co-purified editosome proteins were less abundant in the FL minus β6 truncation, compared to the OB-fold and full length KREPA4 constructs (Figure 4B, top panel). The possibility that other truncations might not have been able to associate with the editosomes owing to their low level of expression, can be ruled out because the FL minus β6 truncation with a similar low expression level in both the total lysate and TEV eluate fractions, was able to incorporate into the editosomes. Overall, the constructs FL minus β6 and OB-fold, which possessed the common OB-fold domain, were able to assemble into the editosome complexes. Other truncations (except OB-fold (C-terminal) construct), which retained the α-helix region of the OB-fold, maintained a direct interaction with free KREPA6 protein, even though these truncations failed to incorporate into the editosomes.

#### (ii) Western blot analysis of calmodulin column eluates

The TEV eluate from each construct was subjected to the second calmodulin column purification step to recover more pure and most stably associated complexes. For each construct, four fractions were eluted from the calmodulin column and eluates 2 and 3, which contained the maximum concentration of proteins, were pooled and subjected to western blot analysis with MAbs against KREPA1, KREPA2, KREPA3 and KREL1, to verify the presence of editosome complexes (Figure 4C, top panel). As observed with the TEV eluates, FL minus β6 and OB-fold constructs were the two truncations besides the full length KREPA4, which continued to retain the major interactions with the editosome complexes upon further purification. This clearly demonstrates that the predicted OB-fold is the domain required for stable association of KREPA4 with the editing complexes. As discussed previously, the editosome proteins were more abundant in the calmodulin column fractions of the full length and OB-fold constructs, in comparison to the FL minus β6 construct. This was again consistent with the level of the tagged proteins in the starting material and the TEV eluates of these truncations. Overall, our data from the calmodulin fractions indicate that the predicted OB-fold is required for integration of KREPA4 in the 20S editosomes.

Western blot analysis of the calmodulin column eluates with antibody against KREPA6 showed that the full length KREPA4, FL minus β6 and OB-fold constructs retained their expected interaction with KREPA6 (Figure 4C, middle panel). However, the truncations FL minus β5 & 6 and FL minus β4, 5 & 6 which showed a possible direct interaction with free KREPA6 in the first purification step (TEV eluate) (Figure 4B, middle panel), could not retain the association in the second step of purification (calmodulin column eluates) (Figure 4C, middle panel). This could be due to loss of the freely occurring KREPA6, whose transient association with these truncations in the first step could not be sustained in the calmodulin purification step, which selects for the most stable interactions.

To detect all the KREPA4 truncations, an antibody against the calmodulin binding protein (CBP) was used to probe the calmodulin column eluates. The amounts of the tagged KREPA4 truncations were again proportional to their levels of expression in the starting material and TEV eluates (Figure 4C, bottom panel). Except the full length KREPA4 and OB-fold constructs, the amounts of all other truncations recovered in the calmodulin column eluates are similarly low, yet FL minus β6 construct is able to integrate into the editosomes. This indicates that the failure of other truncations to associate with the editosome complexes was due to lack of the requisite domains, and not due to the low expression levels of the tagged proteins. Hence, on the basis of the experiments described above, the predicted OB-fold is the domain required for efficient incorporation of KREPA4 into editing complexes.

#### (iii) In-vitro editing assays

**Figure 5:**
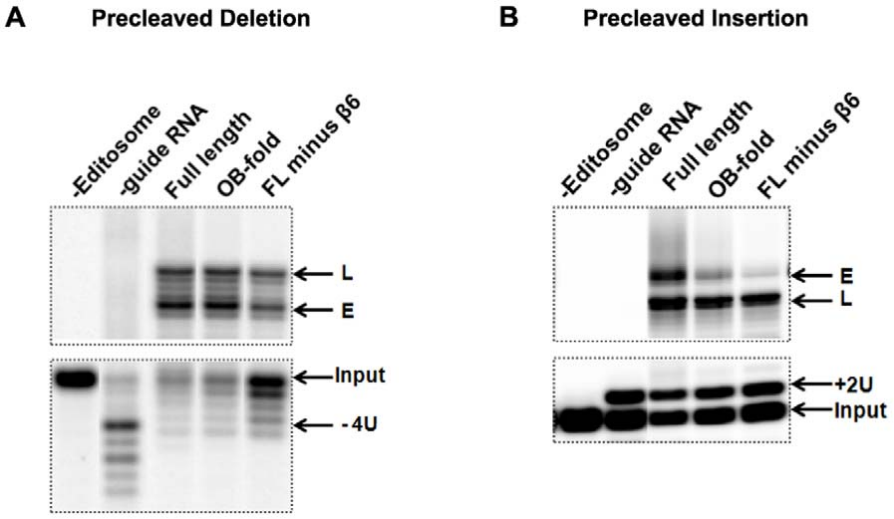
Editosome complexes co-purified with FL minus β6 and OB-fold constructs can catalyze *in-vitro* editing assays. The pooled eluates (2&3) from the calmodulin column were used for in-*vitro* precleaved deletion and insertion editing assays as indicated. The lanes labelled as (-Editosome) and (-guide RNA) represent negative controls lacking either the editosome or the gRNA. *(A)* Precleaved deletion assay; Input RNA with four Us removed (−4U), RNA edited by the deletion of 4 Us (E), and ligated RNAs without any Us removed (L) are indicated. *(B)* Precleaved insertion assay; Input RNA with two added Us (+2U), edited RNA with the insertion of two Us (E), and the ligated RNAs without any Us inserted (L) are indicated.

The calmodulin eluates from FL minus β6 and OB-fold constructs were tested for RNA editing activities using *in-vitro* precleaved U-deletion (Figure 5A) and U-insertion (Figure 5B) editing assays, in which the first step of endonucleolytic cleavage is bypassed by employing a precleaved mRNA substrate. Editing complexes co-purified with both truncations showed the U insertion/deletion and ligation activities present in full length KREPA4 tagged complexes. The level of enzymatic activities were consistent with the amount of editosome proteins co-purified with the tagged truncations, full length KREPA4 and OB-fold associated editing complexes being more active than FL minus β6 associated complexes. Overall, not only do these truncations associate with the editosomes, but they also seem to retain the interactions with the core complex necessary for a functional editosome.

### Incorporation of KREPA4 into Editosomes is not Dependent on its Interaction with gRNA

**Figure 6:**
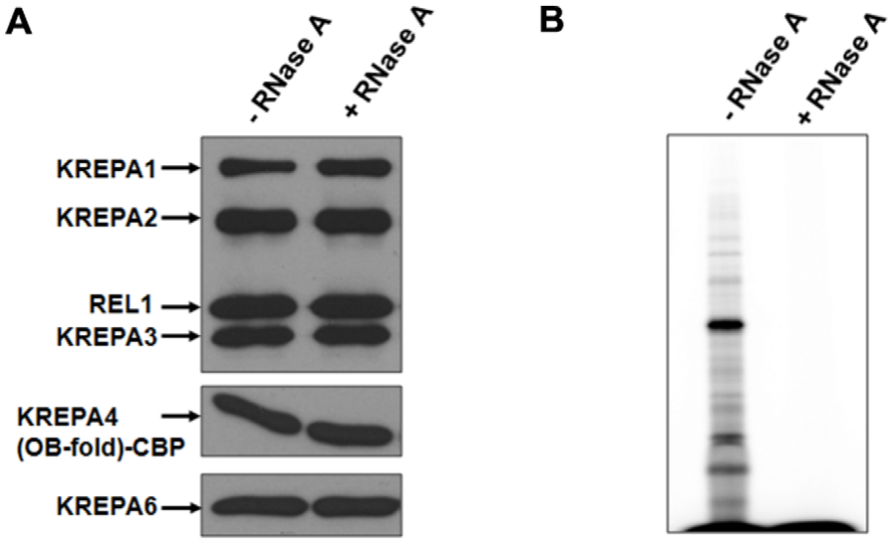
The association of KREPA4 with editing complexes is not mediated via RNA interactions. *(A)* The TEV eluate from KREPA4 (OB-fold) TAP-tagged cells was either treated with RNase A or left untreated, followed by calmodulin affinity purification and western analysis of eluates with antibodies as indicated. (B) pCp labelled RNA extracted from the calmodulin eluates of RNase treated and untreated samples as indicated, to monitor RNase degradation.

While the predicted α-helix of KREPA4 OB-fold mediates the interaction with KREPA6, the TAP-tag experiments clearly demonstrate that it is not sufficient for incorporation of KREPA4 into editosomes, for which the entire OB-fold domain is required. The interaction of KREPA4 with gRNAs is also mediated by the OB-fold [46], and therefore the role of RNA in linking KREPA4 with editing complexes was examined. We wanted to investigate whether the entire OB-fold is required for additional protein-protein interactions, or is it crucial for integration of KREPA4 into the editosome complexes primarily through gRNA interaction. Editosomes were obtained from *T. brucei* cell line expressing TAP-tag of KREPA4 OB-fold construct. After the first TEV purification step, the TEV eluate was split in half and one half was treated with RNase A, while the other half was left untreated. Treated or untreated samples were then bound to the calmodulin column, and the eluted fractions were pooled and subjected to western blot analysis to compare the amounts of editosome proteins in the two samples. As shown in Figure 6A, the interaction of tagged KREPA4 with editosomes remained unaffected by RNase treatment, which effectively degraded all the RNA in the treated sample (Figure 6B). Therefore, the association of KREPA4 with editing complexes is not mediated via RNA interactions, and the OB-fold is possibly involved in other uncharacterized protein interactions.

## Discussion

Initially defined as an oligonucleotide/oligosaccharide-binding module, the OB-fold domain has emerged as a diverse protein fold which can also mediate protein-protein interactions, such that proteins containing these domains often associate with each other to form multi-OB-fold complexes [36], [37]. So is the case with OB-fold containing KREPA proteins, which form a network of interactions, essential for the stability and functioning of the 20S editosomes [38]. In this study we have localized the KREPA4 domain and/or region thereof, for interaction with the 20S editosomes and KREPA6. Our data show that the predicted α-helix of the KREPA4 OB-fold provides the interface for specific interaction with KREPA6, whereas the entire OB-fold domain is responsible for stable integration of KREPA4 in 20S editosomes.

Schnaufer et al had reported a direct interaction between full-length KREPA6 and KREPA4^26–218^ using a yeast two-hybrid screen [38]. We have mapped the interaction interface to KREPA4 residues 153–169 which comprise the α-helix between β strands 3 and 4 of the predicted OB-fold. Our result finds precedent in a study reporting the crystal structure of the N-terminal OB-fold of yeast telomere protein Cdc13 (Cdc13_OB1_), in which a similar α-helix between strands β3 and β4 of Cdc13_OB1_ (αB helix) has been shown to participate in dimerization [50]. In the dimeric conformation of Cdc13_OB1,_ the αB helix and strand β5 from one subunit form a hydrophobic groove that accommodates the αB helix from the other subunit. Hence the role of this α-helix in protein-protein interactions of OB-fold proteins is well-documented.

The recently reported crystal structure of the KREPA6-KREPA3 heterodimer in complex with two copies of a cross-reacting nanobody shows that the residues forming the interaction interface in the KREPA6-KREPA3 heterodimer, mainly span across the β-strands and loops of the two OB-fold domains [51]. The KREPA6 homodimer also shares a similar general architecture [52]. In contrast to our data, none of the contact points in either the KREPA6-KREPA3 heterodimer or the KREPA6 homodimer are localized on the α-helix connecting β strands 3 and 4 of the OB-folds. However, it must be noted that while KREPA6 OB-fold shares only 18.5% sequence identity with the OB-fold of KREPA4, it shares ∼40% sequence identity with the OB-fold of KREPA3 [52], so much so that the anti-A3 nanobody was reported to cross-react with KREPA6 in the crystal structure of the KREPA6-KREPA3 heterodimer [51]. This might explain the difference in the mode of interaction of KREPA6 with KREPA3 versus KREPA6 with KREPA4.

The interaction with KREPA6 is the only known direct interaction of KREPA4 with another editosome protein. KREPA6 is central to the integrity of the 20S editosomes, owing to its multiple interactions with other KREPA family proteins as well as gRNA and mRNA [38], [44]. Hence, we wanted to investigate whether or not the interaction with KREPA6 is the only interaction by means of which KREPA4 gets incorporated into the 20S editosomes. We found that this seemed not to be the case, because the results of our TAP-tag experiments with full length and truncated KREPA4 constructs indicated that the entire predicted OB-fold domain was essential for stable integration of KREPA4 in 20S editosomes. On the other hand, while the truncations retaining the α-helix region of the KREPA4 OB-fold (FL minus β5 & 6 and FL minus β4, 5 & 6) were able to interact with KREPA6 protein, they failed to integrate into the editosome complexes (Figure 3B, top and middle panel). This is likely due to the absence of a complete OB-fold domain in these constructs. Hence, in addition to the direct interaction with KREPA6, there seem to be other interactions which facilitate the incorporation of KREPA4 into editosome complexes. The OB-fold mediated interaction of KREPA4 with gRNA [46], however, seems not to play a role in the integration of KREPA4 into 20S editosomes, as this association is not RNase sensitive. Therefore, there might be other, uncharacterized protein-protein interactions of KREPA4 which mediate incorporation into editosomes or possibly, binding with gRNA and/or KREPA6 induces a conformational change in KREPA4 which in turn forges other new interactions with editosome proteins. The FL minus β6 construct, which lacks the sixth β strand of the predicted OB-fold, was able to assemble into the editosomes, inspite of a truncated OB-fold domain. Probably, this part of the OB-fold is not involved in any major interaction with the editing complexes and is therefore dispensable.

Our finding that the OB-fold domain is essential in assembling KREPA4 into the editosome complexes is consistent with an earlier study reporting that the OB-fold of KREPA3 is critical for the protein interactions of KREPA3 in the editosome, because tagged KREPA3 with the OB-fold deleted does not incorporate into editosomes nor rescue 20S editosomes upon loss of wild-type KREPA3 [43]. Here, in this report, not only have we defined the KREPA4 region that forms the interface for direct interaction with KREPA6, but have also identified the OB-fold as the domain crucial for the efficient incorporation of KREPA4 in the 20S editosomes. In the absence of any structural data for KREPA4, our results convincingly corroborate the presence of, and experimentally validate the boundaries of the KREPA4 OB-fold, as predicted in a previous study [12].

**Figure 7:**
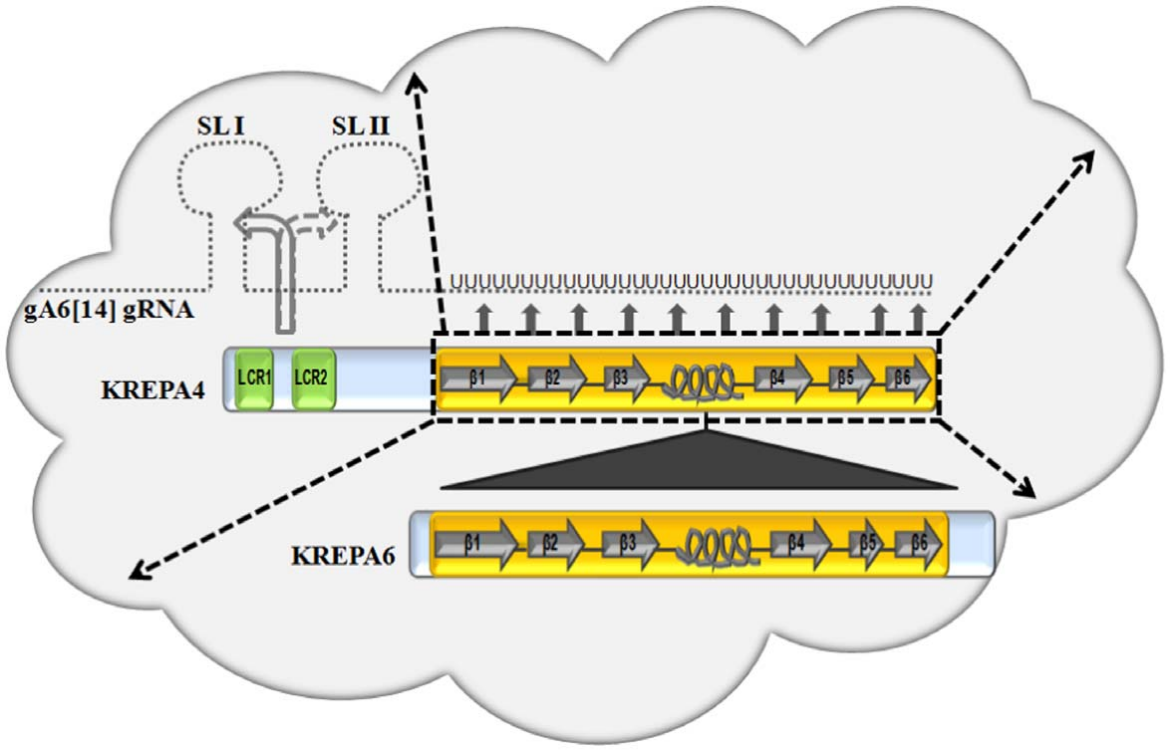
Proposed model for the RNA and protein interactions of KREPA4 in the 20S editosomes. The editosome complex is depicted in the form of a cloud. Full length KREPA4 and KREPA6 proteins are shown as a part of the complex. The secondary structure of their OB-folds is represented schematically. Based on the current *in vitro* studies, the α-helix of the KREPA4 OB-fold is shown to interact directly with KREPA6 (filled triangle), while the entire OB-fold is shown to form stable associations with the editosome complex (thick, dashed arrows). In addition, based on previous *in vitro* RNA binding studies, the OB-fold of KREPA4 also mediates a high-affinity binding to the gA6 [14] gRNA oligo(U) tail (filled arrows), and this contact is stabilized by the LCRs through sequence-specific interactions with the guide stem–loop elements (curved arrows) [46]. The interaction of KREPA4 with stem–loop I (SL I) is preferred over that with stem–loop II (SL II) and is indicated by a solid curved arrow versus a dashed curved arrow, respectively. The oligo (U) tail of the gRNA is extended to fit the model.

Based on the current study, combined with our earlier model for the interaction of KREPA4 with gRNA *in vitro* [46], here we describe a model for the RNA and protein interactions of KREPA4 in the 20S editosomes (Figure 7). The α-helix of the KREPA4 OB-fold forms the interaction interface with KREPA6, while the intact OB-fold is required to form stable associations with the editosome complex. The OB-fold domain also mediates a high-affinity binding to the gRNA oligo(U) tail *in vitro*, and this contact is stabilized by the LCRs through sequence-specific interactions with the guide stem–loop elements. The nucleic acid-binding interface of the great majority of OB-fold proteins is centered on β strands 2 and 3, and this interface is greatly augmented by the variable loops connecting the β strands. We postulate that the RNA recognition surface of KREPA4 OB-fold, in a similar manner, is extended across the β strands and the connecting loops. This is based on our previously reported binding affinities of various KREPA4 deletion mutants, containing intact or truncated OB-fold domain [46]. Since every truncation retained one or the other part of the extended RNA recognition surface, hence, the binding affinities of all the deletion mutants were within close range of each other. Therefore, while the predicted OB-fold of KREPA4 seems to interact with gRNA through an extended binding surface spanning the β strands and the loops, the direct interaction with KREPA6, seems to be mediated by the predicted α-helix between β strands 3 and 4 of the OB-fold. Together, both these interactions mediated by separate parts of the same OB-fold, render this domain crucial for structural integrity of editing complexes.

## Materials and Methods

### Construction of Plasmids

pET30a expression vector (Novagen) was used to construct the N-terminal 6X His-tagged full length KREPA6. KpnI/NotI DNA fragment was generated by PCR and inserted into KpnI/NotI digested pET30a vector. The primer pairs used to generate the DNA fragments for cloning into pET30 and all other vectors are summarized in Table 1.

**Table 1:** Forward (F) and reverse (R) primer pairs used for cloning KREPA6 or KREPA4, full length or deletion variants in the indicated vectors.

Vector-Insert	Primer pairs
**pET30-a vector, KREPA6 full length**	**F** 5′CGTCCGGTACCATGCTAGCTCTTACA 3′**R** 5′TAAATGCGGCCGCATTACGATGGCACACC 3′
**pRSET-C vector, KREPA4 constructs**	
Full length	**F** 5′ATCATGGATCCATATGCGGGTGCGTTCA 3′**R** 5′CGGAATTCCGTTAACACTCCAACTCCTGC 3′
FL minus LCR1	**F** 5′CGGGATCCCGTCAGAAACTACTGCCTTCAG 3′**R** 5′CGGAATTCCGTTAACACTCCAACTCCTGC 3′
FL minus both LCRs	**F** 5′GTGACCTGGATCCGAACAAAATGTGACGACAGGAACGGA 3′**R** 5′CGGAATTCCGTTAACACTCCAACTCCTGC 3′
OB-fold	**F** 5′ATCATGGATCCATCCAACTGTGACG 3′**R** 5′CGGAATTCCGTTAACACTCCAACTCCTGC 3′
OB-fold (C-terminal)	**F** 5′AATATGGATCCATTGGCGGCCAGGA 3′**R** 5′CGGAATTCCGTTAACACTCCAACTCCTGC 3′
OB-fold (N-terminal)	**F** 5′ATAATGGATCCTAGGAGAGCTCGTG 3′**R** 5′ATAGCTGAATTCACGGACCGCTAAG 3′
FL minus OB-fold (C-terminal)	**F** 5′CAGTAGGATCCATTCCGGTATGCT 3′**R** 5′ATAGCTGAATTCACGGACCGCTAAG 3′
FL minus β6	**F** 5′ATCATGGATCCATATGCGGGTGCGTTCA 3′**R** 5′ACGTTGAATTCTGTTAGGGGAGCACCAGAGA 3′
FL minus β5 & 6	**F** 5′ATCATGGATCCATATGCGGGTGCGTTCA 3′**R** 5′CCGCTGAATTCTGTTACGAATCAAAGCATTC 3′
FL minus β4, 5 & 6	**F** 5′ATCATGGATCCATATGCGGGTGCGTTCA 3′**R** 5′ACGCTGAATTCTGTTAGACCAGTCGCTCCAT 3′
KREPA4 mutant 1 (WRPGS)	**F** 5′TTCCCTTAGCGGTCCGTGCAGCAGCAGCAGCAGAAGTTGCTCAGAAT 3′**R** 5′ATTCTGAGCAACTTCTGCTGCTGCTGCTGCACGGACCGCTAAGGGAA 3′
KREPA4 mutant 2 (NRMDEWQK)	**F** 5′AAGTGAAGTTGCTCAGGCAGCAGCAGCAGCAGCAGCAGCAGAAATGGAGCGACT 3′**R** 5′AGTCGCTCCATTTCTGCTGCTGCTGCTGCTGCTGCTGCCTGAGCAACTTCACTT 3′
KREPA4 mutant 3 (EMER)	**F** 5′GATGAGTGGCAAAAGGCAGCAGCAGCACTGGTCGGGCGGCGG 3′**R** 5′CCGCCGCCCGACCAGTGCTGCTGCTGCCTTTTGCCACTCATC 3′
**pLew79-TAP vector, KREPA4 constructs**	
FL minus β6	**F** 5′TGATGAAGCTTATGCGGGTGCGTTC 3′**R** 5′ATAAATGGATCCGGGGAGCACCAGAGA 3′
FL minus β5 & 6	**F** 5′TGATGAAGCTTATGCGGGTGCGTTC 3′**R** 5′TGTCACGGATCCCGAATCAAAGCATTC 3′
FL minus β4, 5 & 6	**F** 5′TGATGAAGCTTATGCGGGTGCGTTC 3′**R** 5′ATAATGGATCCGACCAGTCGCTCCAT 3′
FL minus OB-fold (C-terminal)	**F** 5′TGATGAAGCTTATGCGGGTGCGTTC 3′**R** 5′TGATGGGATCCACGGACCGCTAAGGG 3′

pRSET-C expression vector (Invitrogen) was used to construct the N-terminal 6X His and Xpress-tagged full-length KREPA4 and its truncated or mutated forms. BamHI/EcoRI DNA fragments were generated by PCR and inserted into BamHI/EcoRI digested pRSET-C vector. Mutagenesis of residues in the α-helix of KREPA4 OB-fold; was performed by using a Quick-change lightning site directed mutagenesis kit (Agilent), as per the manufacturer’s protocol. The primer pairs used for mutagenesis are also summarized in Table1.

pLew79-TAP vector [53] was used to construct the C-terminally TAP-tagged KREPA4 truncations. HindIII/BamHI DNA fragments were generated by PCR and inserted into HindIII/BamHI digested pLew79-TAP vector. For the TAP-tagged OB-fold and OB-fold (C-terminal) constructs, in which the MIS was fused to these fragments, gene synthesis was done by Genscript, USA.

### Co-immunoprecipitation (CoIP) Experiments

Unlabelled KREPA6 and KREPA4 proteins were expressed in a cell free, coupled transcription-translation system (TNT) as specified by the manufacturer (Promega) and run on a SDS-PAGE gel alongside Smart-His tagged protein standards (Genscript). This was followed by western blotting with anti-6X His monoclonal antibody to enable quantification of the His-tagged proteins using versadoc (Biorad).

For the CoIP of KREPA6 in the presence or absence of various KREPA4 variants, ^35^S-labelled KREPA6 and unlabelled KREPA4 proteins were expressed with the TNT system. Equimolar concentrations of the proteins (1 pmole) were mixed and incubated for 30 min at room temperature. Dynabeads M-280 Sheep anti-Mouse IgG (Invitrogen); 5 µl per reaction, were coupled with Anti-Xpress Antibody (Invitrogen); 0.5 µl per reaction, in IP200 buffer (10 mM Tris pH 7.4, 10 mM MgCl2, 200 mM KCl, 0.1% Triton X-100 and 1% BSA). These were incubated for 1 h at 4°C with bi-directional mixing and then washed two times with IP200 buffer. Protein mixtures were incubated with antibody-coated beads in IP200 buffer for 15 min at 4°C with rotation. The beads were washed three times with IP200 buffer and resuspended in 1X SDS loading dye followed by SDS PAGE and visualization of interactions by phosphorimaging. To demonstrate the pull-down of KREPA4 proteins, essentially the same experiment as described above, was carried out with ^35^S-labelled KREPA4 variants and unlabelled KREPA6 protein.

The polyclonal anti-KREPA4 antibody, raised against a synthetic peptide sequence of KREPA4, was affinity purified from rabbit antisera by standard procedures. The pre-immune serum was carried through the same purification procedures. In case of the CoIP with anti-KREPA4 antibody (Figure 2B), 1 pmole of KREPA4 protein expressed with the TNT system, was first incubated with ∼3 pmoles of the purified anti-KREPA4 antibody or the pre-immune serum for 10 min at room temperature. This was followed by addition of equimolar concentration of KREPA6 and further incubation for 30 min at room temperature. Subsequent steps were the same as described above.

The two peptides; Peptide#1 (RPGSEVAQNRMDEWQKEMERLVDLYDDDDK) and Peptide#2 (DDRNGELVRGRIQFASRCAEGPDLYDDDDK) were synthesized by Genscript, USA. The underlined sequence corresponding to the Xpress tag, enabled pull down of the two peptides by Dynabeads M-280 Sheep anti-Rabbit IgG (5 µl per reaction), coupled with Anti-DDDDK-tag antibody (Genscript; 1 µl per reaction). 1 µg of each peptide was incubated for 30 min at room temperature, with 250 ng of ^35^S-labelled KREPA6, expressed with the TNT system. Subsequent steps were the same as described above. The immunoprecipitated peptides were detected by western blotting with Anti-DDDDK-tag antibody (Genscript).

### Transfection, Purification of TAP-tagged Proteins and Western Blotting

The *T. brucei* PF 29-13 cell line which co-expresses the Tet repressor protein and T7 RNA polymerase [54] was transfected with 10 µg of NotI-linearized pLew79-TAP plasmid, each one encoding a different KREPA4 construct. The resulting stable cell lines were selected by growth in SDM-79 medium containing 10% fetal bovine serum and supplemented with 15 µg/ml of G418, 25 µg/ml of hygromycin, and 2.5 µg/ml of phleomycin. Expression of the tagged proteins was induced by adding 100 ng/ml of tetracycline. Tagged KREPA4 complexes were purified from 2L of cells as described before [53]. The whole-cell lysates, TEV eluates and calmodulin column eluates from each KREPA4 construct were resolved by SDS–PAGE and assayed accordingly by Western blotting with PAP reagent, MAbs against KREPA1, KREPA2, KREPA3, and KREL1 and polyclonal antibodies against KREPA6, KREPA4 and CBP-tag.

To test the effect of anti-KREPA4 antibody on the stability of editosomes, tagged KREPA4 complexes were purified from 2L of cells as described [53]. The TEV eluate obtained after digestion with TEV protease was divided into two equal parts. One part was incubated with the purified anti-KREPA4 antibody (∼2 µg), and the other with the pre-immune serum, which was carried through the same purification procedures. In the presence of protease inhibitors and RNasin, the protein-antibody mixtures were incubated for 2 hrs at 4°C with rotation, followed by binding to the calmodulin affinity beads overnight at 4°C with rotation. Fractions eluted from the calmodulin column were pooled and assayed by Western blotting with MAbs against KREPA1, KREPA2, KREPA3, and KREL1, and polyclonal antibodies against KREPA6, KREPA4.

To investigate the RNase sensitivity of KREPA4 association with editosomes, complexes were purified from 2L of cells expressing the TAP-tagged KREPA4 OB-fold protein. One half of the TEV eluate was incubated with 0.1 mg/ml RNase A for 12 hrs at 4°C with rotation, while the other half was left untreated. This was followed by binding of the treated and untreated samples to the calmodulin affinity beads for 4 hrs at 4°C with rotation. Fractions eluted from the calmodulin column were pooled and assayed by Western blotting with antibodies as described above. In order to monitor the RNase A degradation, total RNA was extracted from the treated/untreated calmodulin eluates, pCp labelled and run on a 9% polyacrylamide gel containing 7 M urea.

### *In vitro* RNA Editing Assays

The substrates for a precleaved insertion assay (5′CL18 and 3′CL13pp with gPCA6-2A RNAs), specifying the insertion of two U’s were prepared as described previously [55]. The substrates for a precleaved deletion assay (U5 5′CL and U5 3′CL with gA6 [14]PC-del RNAs), specifying removal of four U’s were prepared as described previously [56]. 5′CL18 and U5 5′CL were 5′ labeled with [^32^P]ATP by T4 kinase. All RNAs were purified by gel electrophoresis on a 9 or 15% denaturing polyacrylamide gel containing 7 M urea. Calmodulin eluate fractions 2 and 3 from TAP-tag purification of full length or truncated KREPA4 proteins were pooled and 10 µl was incubated with the different substrate RNAs for enzymatic reactions. An equal volume of 7 M urea dye was added to all samples, which were run on a 9 or 15% polyacrylamide gel containing 7 M urea, according to their sizes and visualized by phosphorimaging.

### Indirect Immunofluorescence

PF trypanosomes, uninduced or induced to express the TAP-tagged proteins were grown to mid-logarithmic phase. ∼ 2×10^8^ cells were spun down, resuspended in fresh media containing 150 nM Mitotracker Red CMXRos (Invitrogen) and incubated at 28°C for 45 min. Cells were spun down again and washed once in TDB (20 mM Na2HPO4, 2 mMNaH2PO4, 5 mM KCl, 80 mM NaCl, 1 mM MgSO4, 10 mM glucose [pH 7.4]), resuspended to 6–7×10^6^ cells/ml in TDB, and spotted onto coverslips coated with poly-L-Lysine (Sigma). All steps were carried out at room temperature. The parasites were allowed to adhere to the coverslips for 1 h and then fixed for 10 min with 4% paraformaldehyde in PBS. After washing twice with PBS, cells were permeabilized by treatment with 0.2% Triton X-100 in PBS for 15 min. After washing twice with PBS again, cells were blocked for 1 h with 3% BSA in PBS. This was followed by incubation for 1 h, with Rabbit Anti-Protein A antibody (P3775, Sigma), which was diluted 1∶40,000 in PBS. After washing three times with PBS, the cells were incubated for 1 h in a moist chamber with Alexa Fluor 488 Goat Anti-Rabbit IgG (Invitrogen), which was diluted 1∶500 in PBS containing 3% BSA. After washing three times in PBS, the cells were then treated for 15 min with a 2 µM DAPI solution in PBS to stain DNA. The cells were washed three times with PBS and mounted with Fluoromount-G (Southern biotech). Fluorescence was observed with a Nikon epifluorescent microscope equipped with the appropriate filters.

### Gel Shift Assay

The gA6 [14] guide RNA used in this assay was prepared by T7 polymerase (Promega) transcription of PCR-generated templates as described previously [8]. The wild-type or mutated KREPA4 proteins were expressed with the TNT system, and equimolar concentrations (∼1.5 pmoles) of both were pulled down with Dynabeads for His-tagged protein isolation (Invitrogen), according to manufacturer’s protocol. The proteins were eluted with a low pH buffer (50 mM Glycine pH 2.4) and immediately neutralized with 500 mM Tris pH 9. Gel shift assay was performed by incubating the proteins with 3′ end labelled gA6 [14] gRNA in GS-RBB50 buffer as described previously [45].

### Solvent Accessibility Predictions

The solvent accessibility prediction for KREPA4 was obtained with I-TASSER, available as a public web server at http://zhanglab.ccmb.med.umich.edu/I-TASSER [57].
